# Phage Eco-Locator: a web tool for visualization and analysis of phage genomes in metagenomic data sets

**DOI:** 10.1186/1471-2105-12-S7-A9

**Published:** 2011-08-05

**Authors:** Ramy K Aziz, Bhakti Dwivedi, Mya Breitbart, Robert A Edwards

**Affiliations:** 1 Department of Computer Science, San Diego State University, San Diego, CA, 92182, USA; 2 Department of Microbiology and Immunology, Faculty of Pharmacy, Cairo University, Cairo, Egypt; 3 College of Marine Science, University of South Florida, St. Petersburg, FL, USA; 4 Mathematics and Computer Science Division, Argonne National Laboratory, Argonne, IL, USA

## Background

Bacteriophages, viruses that infect bacteria, are the most abundant biological entities on our planet, and their nucleic acids constitute a substantial proportion of total DNA in Earth's ecosystems [1,2]. While the advent of metagenomic methods has allowed the rapid and efficient investigation of microbial and viral communities [3-5], there has not been a comprehensive comparative analysis of phage genes and genomes present in all sequenced ecosystems [6,7]. To examine the abundance and distribution of phage genes in environmental metagenomic sequences, we developed a web-based tool, Phage Eco-Locator [http://www.phantome.org/eco-locator] that screens all publicly available sequenced metagenomes for a user-defined phage genome, or all phage genomes within a user-selected metagenomic sample.

## Materials and methods

**Figure 1:**
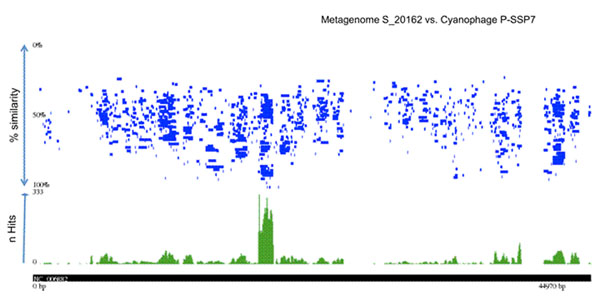
**An example of Eco-Locator output.** The ***upper panel*** is a scaffolding plot (recruitment plot) where each metagenomic sequence read (horizontal blue bars) is mapped to its exact location within a phage genome. The Y-axis of the upper panel represents the average percent similarity of metagenomic sequence read to the target region in the phage genome. The ***lower panel*** represents a map of every nucleotide in the phage genomes that hits a metagenomic sequence read. The Y-axis of the lower panel represents the number of hits per genomic position.

The tool relies on pre-calculated tBLASTX searches in which metagenomic sequence reads are the input query and all phage genomes are the BLAST database [8]. For optimization, several BLAST parameters have been tested, and the best results are obtained when all tBLASTX matches above a threshold E-value of 10^-5^ are included as positive hits. Positive hits are then mapped to phage genome scaffolds and visualized in two different types of plots: one representing sequence hits at different similarity scores (Fig. 1; upper panel) and another representing the coverage density over phage nucleotides (Fig. 1; lower panel).

## Results

All 588 phage genomes available in the PhAnToMe database [http://www.phantome.org] (as of January 1, 2011) were screened against 296 de-replicated metagenomic libraries. The graphical output was translated into metrics representing phage abundance, extent and breadth of distribution, and coverage density and evenness. Applying these metrics to all samples demonstrated a pervasive, yet uneven, distribution of phage genes in metagenomic libraries and allowed the separation of phage genomes into distinct groups. The analyses also showed a tendency for phage genomes to prevail in environments similar to their original isolation source, where their bacterial hosts are expected to thrive (e.g., cyanophages in aquatic samples and halophages in hypersaline environments).

## Conclusion

Phage Eco-Locator effectively allows the global analysis of all phage sequences in metagenomes while also permitting gene-level analysis of individual phage genomes. In the future, application of this tool to sequences from a wide range of ecosystems will enhance our understanding of the factors controlling phage biogeography and environmental selection.
